# Observations on a Disease of the Bones, Known by the Name of Necrosis

**Published:** 1782

**Authors:** 


V. *Obſervations ſur une Maladie d'Os, connüe ſous le nom de Necroſe.* i. e. *Obſervations on a Diſeaſe of the Bones, known by the name of Necroſis. By* M. David, M. D. *Regius Profeſſor of Anatomy and Surgery, Member of the Academy of Sciences, and principal Surgeon to the Hotel Dieu at Rouen.* 8vo. Paris, 1782. 28 pages.

M. BRUN, Surgeon at Toulouſe, in a piece entitled '*Memoire pour les pauvres* '*malades de l'Hotel Dieu de Toulouſe,*' it ſeems, has controverted the opinion that a new bony cylinder may be formed round an old bone. He aſſerts that no caſe of this ſort is to be met with either in Ruyſch or Scultetus; and after giving it as his opinion that what has been conſidered as a new bone, is nothing more than a general exoſtoſis of the old one, accompanied with internal caries, he condemns the operation practiſed in ſuch caſes by M. David, as cruel, dangerous, and uſeleſs. The pamphlet before us contains M. David's reply to this cenſure.

He begins with appealing to the teſtimony of Meſſieurs Duhamel, Bordenave, and Troja. Theſe gentlemen, we are told, have not only ſeen a variety of inſtances where a long bone has been found loose and inclosed in a bony case, but have even succeeded in producing this phænomenon in a living animal at pleasure *. We may add from our own knowledge, that specimens of the disease in question are not unfrequent in anatomical collections.

M. David observes, that in several cases he has extracted considerable portions of bone, which formed complete cylinders of the humerus, cubitus, tibia, and even femur. Some of these were an inch and a half thick, and had a number of fistulous openings, several inches distant from each other.

This disease, which at first sight appears so extraordinary, is truly a disease of the periosteum, and has sometimes been brought on by a violent blow, and even merely by sleeping on damp ground. If this membrane is contused, or irritated either by external means or by an acrid humour, so as to occasion a suppuration, it will be gradually separated from the bone, and the matter by degrees making its way through the periosteum to the cellular membrane, the bone, to a certain extent, will become a dead substance. But during all this time the periosteum continu-

* See page 357.

ing to receive the offific juices, a new bone is gradually formed, and the openings in the periofteum become fo many bony fiftulæ.

Sometimes, we are told, the pus, inftead of making itfelf little vents through the periofteum, deftroys this membrane in form of a rent, *(en forme de dechirure)* and then, as in the cafes defcribed by Ruyfch and Scultetus, the integuments are fooner affected and diftended, and when the abfcefs is opened, a loofe bone may be felt within a new bony production. This is faid to happen moft frequently in the tibia, where it is covered only by integuments.

M. David tells us, he is now able to produce upwards of fifteen inftances of this difeafe that have occurred in his own practice. The moft recent and the moft fingular of thefe happened in November 1781. In this cafe there was a portion of the os femoris, feven inches in length, completely furrounded by a new bony cylinder, eight tenths of an inch thick, and almoft as hard as the original bone. Two fiftulous openings, one of them a little below the great trochanter, the other at the lower and outer part of the thigh, concurred, with the increafed bulk of the thigh, to convince our author of the nature of the difeafe. Thefe figns, he adds, have never deceived him.

It is perhaps hardly poffible for the art of furgery to produce any thing more ftriking than the operation he performed in this cafe. He began with removing integuments, fafcia lata, and mufcles, in a fpace of about ten inches in length, and four or five in breadth, fo as to lay bare this new bony mafs, and to be able with a chifel, gouge, and mallet, to make an opening in it fufficient to extract the dead bone. This operation, he affures us, was followed by no alarming fymptom. All the other cafes, he adds, have fucceeded equally well, and he now does not even find it neceffary to confine the patient to a very low regimen.

